# Autoresuscitation: A Case and Discussion of the Lazarus Phenomenon

**DOI:** 10.1155/2015/724174

**Published:** 2015-06-08

**Authors:** Kjartan Eskjaer Hannig, Rasmus Wulff Hauritz, Erik Lerkevang Grove

**Affiliations:** ^1^Department of Anaesthesiology, Kolding Hospital, Skovvangen 2-8, 6000 Kolding, Denmark; ^2^Department of Cardiology, Aarhus University Hospital, Palle Juul-Jensens Boulevard 99, 8200 Aarhus, Denmark

## Abstract

Lazarus phenomenon or autoresuscitation is a very rare condition defined as delayed unassisted return of spontaneous circulation after cessation of cardiopulmonary resuscitation. Based on a case with a 67-year-old male who came back to life after discontinuation of cardiopulmonary resuscitation, we discuss the background and possible countermeasures related to the Lazarus phenomenon.

## 1. Introduction

Lazarus phenomenon or autoresuscitation (AR) is a very rare condition defined as delayed unassisted return of spontaneous circulation (ROSC) after cessation of cardiopulmonary resuscitation (CPR) [1, 2]. After being first reported by Linko et al. in 1982 [3], it was later termed the “Lazarus phenomenon” by Bray Jr. in 1993 [4] after the biblical figure Lazarus, whom Jesus supposedly resurrected four days after his death and burial (Gospel of John Chapter 11: 1–44).

The occurrence of this phenomenon may be widely underreported as illustrated by the fact that almost 50% of French emergency physicians claim to have encountered AR in clinical practice [5] and by the statement by Dhanani et al. that more than one-third of Canadian intensivists have seen at least one case of AR [6]. The true incidence remains unknown.

## 2. Case Report

**Figure 1:**
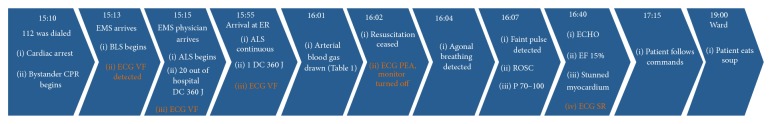
Timeline. CPR: cardiopulmonary resuscitation. EMS: emergency medical services. BLS: basal life support. ECG: electrocardiogram. VF: ventricular fibrillation. ALS: advanced life support. DC: direct current. ER: emergency room. PEA: pulseless electrical activity. ROSC: return of spontaneous circulation. ECHO: echocardiography. SR: sinus rhythm.

A 67-year-old Caucasian male collapsed with cardiac arrest outside his home (Figure 1). A nurse, who was caring for the patient on a daily basis, coincidently passed by and immediately initiated CPR. The emergency medical services (EMS) were called at 15:10 hours and an ambulance arrived at 15:13 and the emergency physician at 15:15. On arrival, the initial rhythm was ventricular fibrillation. Resuscitation was performed according to the advanced life support (ALS) algorithm [7] including chest compression, ventilation, intubation in the field, a total of 20 defibrillations, and standard drug administration, including epinephrine and amiodarone.

The patient had massive comorbidity on the basis of universal atherosclerosis due to 31 pack years of smoking, severe hypertension, hypercholesterolemia, and heredity. In 2004, bilateral in situ bypass surgery optimised blood flow to his lower extremities. He was also diagnosed with left ventricular hypertrophy and systolic and diastolic dysfunction with an ejection fraction (EF) of 40%. In 2006, the patient underwent coronary artery bypass graft surgery, complicated by postoperative ventilator therapy for one month, middle cerebral artery infarction, sternal infection, and organic delirium, which elapsed into a psychotic episode and severe depression. In the last three years, the patient had been depending on haemodialysis three times a week. Furthermore, the medical history included severe chronic obstructive pulmonary disease with a forced expiratory vital capacity of 45%, abdominal aortic aneurism of 5.1 cm, paroxysmal atrial fibrillation, and chronic musculoskeletal pain due to cervical and lumbal spinal stenosis. His medication comprised carvedilol, ramipril, simvastatin, warfarin, inhaled salmeterol/fluticasonpropionat, inhaled tiotropium, pantoprazole, paracetamol, vitamins and, related to his hemodialysis, alfacalcidol, erythropoietin, phosphate-binding drug, and furosemide.

Nearly an hour after the cardiac arrest at 15:55 the patient arrived at the emergency department still in ventricular fibrillation. Upon arrival, he was defibrillated again. At the next rhythm check, the ECG monitor showed pulseless electrical activity (PEA) with very slow bizarre looking complexes. The decision to stop resuscitation efforts was taken at 16.02, and the monitors were turned off. This was also based on appearing information from the patient's medical files that within the previous three years he had repeatedly rejected resuscitation attempts in the case of respiratory or circulatory arrest. Two minutes later, slow agonal (gasping) breathing was seen, and five minutes later a very faint central pulse was detected. Arterial blood gas (ABG) taken in the last minute of resuscitation had now been analyzed and implied severe metabolic acidosis and hypokalemia (Table 1). An echocardiography (ECHO) showed general hypokinesia with an EF of 15% (see Videos 1 and 2 in Supplementary Material available online at http://dx.doi.org/10.1155/2015/724174). The decision not to resume treatment was upheld, whereas palliative care was continued also on suspicion of hypoxic brain damage. Surprisingly, the patient regained consciousness. One hour later, he blinked and squeezed hands on command. Three hours later he sat up in his bed and had some soup (Figure 1).

The patient was considerably unfound with being resuscitated and refused any further treatment. He was not psychotic and had the capability of making his own decisions. Twenty-two hours after the initial cardiac arrest, he died.

## 3. Discussion

Survival from out-of-hospital cardiac arrest remains poor with only 7.6% being alive to discharge from hospital [8]. Predictors of survival are witnessed cardiac arrest (either bystanders or EMS), effective CPR, shockable rhythm, and return of spontaneous circulation (ROSC) in the prehospital setting [8]. Gasping or agonal breathing is seen in approximately 50% of cardiac arrests [9–11]. It is time-limited and lasts approximately 4 min in at least one-third of cases [12]. Survival to hospital discharge is 3 times greater compared with those not found gasping [9, 11].

In a comprehensive review from 2010, 32 cases of AR were identified from 16 different countries over a 26-year period (1982–2008) [1]. The studies were all considered of very low quality (case reports or letters to the editor). In only eight studies, continuous ECG monitoring was reported and, in these, AR did not occur beyond 7 minutes after failed CPR [1].

Another author states that about half of (reported) AR cases achieved good neurological recovery following ROSC and the other half died soon after [2], thus reflecting the point that AR usually occurs in a patient, who has likely experienced irreversible end-organ damage. Some of the patients achieving good neurological recovery died during their hospital stay [2]. Of all patients with (reported) AR, about 35% were eventually discharged home neurologically intact [2].

*This Case Illustrates Three Important Points*

1. Ideally, decisions about whether or not it is appropriate to start CPR should be made in advance [7, 13]. Decisions should be communicated to relatives and all the involved caring persons, both hospital staff and the home care nurses, and if possible it should be written down in an electronic advance directive (“living will”) [7, 13].
2. The decision to stop CPR is a challenging clinical task. In general, CPR should continue as long as shockable rhythm or the other reversible cause for cardiac arrest persists [13, 14]. It is widely accepted that asystole for more than 20 minutes without reversible factors is a reasonable cause for stopping CPR [13]. The decision to stop is based on patient preferences, time before initiation of CPR, primary rhythm, comorbidity (including prognosis of underlying cause of cardiac arrest), and duration of resuscitation. Extended efforts are made with intoxication, accidental hypothermia, and pulmonary embolism treated with thrombolysis. Unconditional prolongation of life should not be the goal in itself, rather to achieve a sufficient quality of life [13].
3. An example of the Lazarus phenomenon with its background and possible countermeasures is discussed below (summarized in Box 1).

The pathophysiological mechanisms for AR are poorly understood [1, 2, 15]. Hyperinflation (in obstructive lung disease), myocardial stunning (in acute myocardial infarction), hyperkalaemia (especially in renal failure), delayed action of drugs, countershock asystole, and unobserved minimal vital signs amongst others have been considered to be the most common mechanisms [1, 2, 15].

Unrecognized dynamic lung hyperinflation can theoretically occur in all patients but is especially seen in patients with obstructive lung disease (chronic obstructive pulmonary disease and asthma). It is probably due to rapid manual ventilation with inadequate time for exhalation, leading to elevated end-expiratory pressure (auto-PEEP) [2]. Auto-PEEP is caused by air trapping, that is, air entering the lungs and being unable to escape. This gradual increase of intrathoracic pressure leads to decreased venous return to the heart (preload) and subsequently low cardiac output and cardiac arrest, even in the presence of a perfusable cardiac rhythm [1, 2, 7]. The physiology of severe auto-PEEP is similar to pericardial tamponade [2]. Decreased venous return may also delay drug delivery to the central circulation, impeding medication action during resuscitation.

*Countermeasures*. Avoid hyperinflation by bag-mask ventilation using an inspiratory time of about 1 second and give only enough volume to produce a visible normal chest rise [7]. If the patient is intubated and connected to a ventilator, the tidal volume is set at 6 mL/kg ideal bodyweight at 10 breaths/minute [7]. If hyperinflation is suspected, intermittent disconnection of the tracheal tube for 10 seconds [15] may relieve air trapping permitting return of venous flow and spontaneous circulation [7]. Dynamic hyperinflation increases transthoracic impedance and with shockable rhythms higher shock energies may be considered [7]. Always consider tension pneumothorax or bilateral pneumothorax in asthma-related cardiac arrest. In skilled hands, lung ultrasound is a faster and more sensitive diagnostic test than chest X-ray [7].

Myocardial stunning is due to ischaemia most often because of infarction [2]. After brief periods of myocardial ischaemia, prolonged myocardial dysfunction can occur, followed by gradual recovery and improvement in cardiac output typically within 2-3 days [7, 15]. 

*Countermeasure*. Percutaneous coronary intervention (PCI) should be conducted as soon as possible, when appropriate.

Hyperkalemia prevents adequate efflux of potassium, and hence the resting membrane potential of the myocytes decreases, leaving the myocardium depolarized and the myocytes refractory to further stimulation (unexcitable). The cardiac conduction is slowed and the heart stops in diastole refractory to resuscitation. Voelckel hypothesized this as a mechanism of delayed ROSC [16]. The myocardium is also extremely sensitive to hypokalemia, which alters cardiac tissue excitability and conduction, and may induce malignant ventricular arrhythmias, resulting in cardiac arrest. 

*Countermeasure*. ABG taken early (or even in the prehospital setting) can confirm suspicion.

Delayed delivery and action of administered medications (like epinephrine) can occur, when drugs are administered through a peripheral vein, as central delivery is slow due to impaired venous return. Presumably, some older cases of AR can be attributed to this mechanism, especially due to escalating doses of epinephrine used according to historic ALS guidelines [2]. 

*Countermeasure*. Use intraosseous line or central line if present.

Transient asystole or PEA following defibrillation of prolonged VF is a well-known phenomenon and occurs in about 60% of patients [2, 17]. Surprisingly, it has a worse prognosis than primary asystole or PEA. This may be related to direct myocardial injury due to electrical current and because the fibrillating myocardium requires more oxygen and faster depletes high-energy phosphate stores [17]. 

*Countermeasure*. Use biphasic instead of monophasic defibrillators, using only energy levels specified by the manufacturer.

Unobserved minimal vital signs likely represent another explanation, when the circulation is present again. Pseudo-PEA is the clinical situation in which contractile activity is occurring, yet it is of minimal magnitude. Often, pulse palpation can be challenging or nearly impossible. In such situations, ECHO easily shows if cardiac contractions are present. 

*Countermeasure*. Use ECHO when available. Consider using capnography during CPR, which serves three purposes. Firstly, it confirms correct placement of the endotracheal tube, when consistent (blunted) waveforms are present beyond the first 6-7 respirations, whereas a “flat capnogram” is indicative of accidental oesophageal intubation [7, 18, 19]. Secondly, it reflects pulmonary blood flow and thus cardiac output, providing the opportunity of monitoring the efficacy of chest compression [7, 18, 19]. In healthy individuals, the normal range for end-tidal CO_2_ is 4.65–6.0 kPa (35–45 mmHg). During CPR, an optimal target for end-tidal CO_2_ has not been established [7], but values as high as possible above 1.35–2.65 kPa (10–20 mmHg) are desirable [18]. According to Kodali et al, the initial end-tidal CO_2_ level under CPR may divide patients into those likely to achieve ROSC (values > 1.35 kPa = 10 mmHg) and those not likely to achieve ROSC (values < 1.35 kPa = 10 mmHg) [7, 18]. Thirdly, a significant abrupt and sustained increase in end-tidal CO_2_ during CPR from initial baseline (e.g., increase exceeding 1.35 kPa = 10 mmHg) may be seen as the first indicator of ROSC. This may precede a palpable pulse [2, 7, 15, 18, 19].

In our case, hyperventilation may be a possible explanation, since the patient's pCO_2_ was about 10 kPa (75 mmHg) normally and measured under CPR 7.6 kPa (57 mmHg), which may be considered as relative hypocapnia. Hypokalemia was present and must have been worse at the time of the cardiac arrest, which was followed by nearly one hour of CPR causing anaerobe metabolism. A possible explanation may be that epinephrine stimulates the sodium potassium pump (Na-K ATPase), causing influx of potassium into the cells. Delayed action of drugs may have been important as well, since the patient arrived with a small bore peripheral venous line, which continued to be used during in-hospital resuscitation. Probably, AR in our case was explained by several concurring mechanisms.

Since death is not an event but a process [2], according to the ALS guidelines, the patient must be observed for a minimum of 5 minutes before confirming death [7]. Since most cases of AR occur within ten minutes, it should be considered to extend this period to 10 minutes with ECG monitoring before certifying death or informing the family [2, 3, 15]. The absence of mechanical cardiac function can be confirmed by absence of central pulse on palpation and absence of heart sounds on auscultation [20]. Supplementary, one or more of the following clinical findings can be used: asystole on ECG, absence of pulsatile flow on intra-arterial line, and absence of contractile activity on ECHO [20]. After 5−10 minutes of continued cardiorespiratory arrest, the absence of pupillary response to light, the absence of corneal reflexes, and absence of any motor response to supraorbital pressure should be confirmed [7, 20, 21].

## Supplementary Material

Supplementary Video 1: Parasternal long axis ECHO view.
Supplementary Video 2: Apical 4-chamber ECHO view.

## Figures and Tables

**Box 1:**
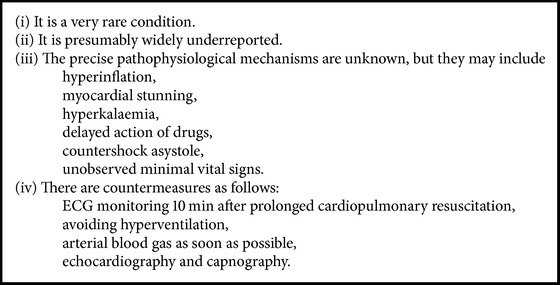
**Box 1: **Facts about the Lazarus Phenomenon.

**Table 1:** Arterial blood gas.

Arterial blood gas		Ref. range
pH	7.01	7.35–7.45
Base excess	−17.9	−3.0–3.0
Hydrogencarbonate	11.4 mmol/L	22.0–27.0
Lactate	17.0 mmol/L	0.5–1.6
pO_2_	26.7 kPa	11.1–14.4
pCO_2_	7.6 kPa	4.7–6.4
Hemoglobin	7.8 mmol/L	8.3–10.5
Sodium	136 mmol/L	137–145
Potassium	2.7 mmol/L	3.5–4.4
Calcium-ion-free	1.25 mmol/L	1.18–1.32
Glucose	25.0 mmol/L	
